# Preemptive use of intravenous ibuprofen to reduce postoperative pain after lower third molar surgery: a systematic review of randomized controlled trials

**DOI:** 10.6061/clinics/2021/e2780

**Published:** 2021-06-23

**Authors:** Pedro Urquiza Jayme Silva, Daniela Meneses-Santos, Walbert de Andrade Vieira, Juliana Cama Ramacciato, Ricardo Pedro da Silva, Marcelo Caetano Parreira da Silva, Sigmar de Mello Rode, Luiz Renato Paranhos

**Affiliations:** I Program de Pos-Graduacao em Odontologia, Faculdade de Odontologia, Universidade Federal de Uberlandia, Uberlandia, MG, BR; II Programa de Residencia, Departamento de Cirurgia e Traumatologia Buco-Maxilo-Facial, Faculdade de Odontologia, Universidade Federal de Uberlandia, Uberlandia, MG, BR; III Departamento de Odontologia Restauradora, Divisao de Endodontia, Faculdade de Odontologia de Piracicaba, Universidade Estadual de Campinas (UNICAMP), Piracicaba, SP, BR; IV Departamento de Farmacologia, Anestesiologia e Terapeutica, Faculdade de Medicina e Odontologia e Centro de Pesquisas Odontologicas Sao Leopoldo Mandic, Campinas, SP, BR; V Departamento de Cirurgia e Traumatologia Buco-Maxilo-Facial, Faculdade de Odontologia, Universidade Federal de Uberlandia (UFU), Uberlandia, MG, BR; VI Departamento de Materiais Odontologicos e Protese, Instituto de Ciencia e Tecnologia, Universidade Estadual Paulista Julio de Mesquita Filho, Campus Sao Jose dos Campos, Sao Jose dos Campos, SP, BR; VII Area de Odontologia Preventiva e Social, Faculdade de Odontologia, Universidade Federal de Uberlandia (UFU), Uberlandia, MG, BR

**Keywords:** Intravenous Ibuprofen, Ibuprofen, Third Molar Surgery, Preemptive Analgesia, Pain, Postoperative Pain

## Abstract

This study aimed to systematically review the literature to assess the effect of preemptive intravenous ibuprofen on pain reduction after lower third molar surgery. Nine databases (PubMed, Scopus, LILACS, SciELO, Embase, Web of Science, Cochrane, Open Gray, and Open Thesis) were used as sources of research, including “grey literature.” The protocol was registered in PROSPERO. Only randomized clinical trials evaluating the effects of preemptive intravenous ibuprofen on pain during and immediately after the extraction of lower third molars were included, without restrictions of year and language. Two reviewers independently performed the study selection, data extraction, and assessment of the risk of bias. The “Joanna Briggs Institute for Randomized Controlled Trials” tool was used to assess the risk of bias. Each study was categorized according to the percentage of positive responses to the questions corresponding to the assessment instrument. The results were measured narratively/descriptively. The initial search resulted in 3,257 records, of which only three studies (n=150 participants) met the eligibility criteria and were included in the qualitative analysis. All studies were published in 2019. The risk of bias ranged from low to moderate. Two studies found significant pain reduction within 48 h after the procedure. In conclusion, the use of preemptive intravenous ibuprofen for extracting third molars reduces pain and analgesic consumption after the surgical procedure.

## INTRODUCTION

Surgical removal of the third molars is one of the most common procedures in oral surgery (1). It can cause trauma to soft tissues and the bone, resulting in a postoperative inflammatory process in response to pain, edema, and trismus (2). The control of postoperative pain after the removal of third molars has attracted significant interest in scientific literature (2-4). Preemptive analgesia is the concept of a preoperative antinociceptive approach that prevents or modulates postoperative pain (5,6). Preemptive analgesia using non-steroidal anti-inflammatory drugs (NSAIDs) in third molar surgeries has been effective (7-9).

NSAIDs inhibit the cyclooxygenase (COX), COX-1, and COX-2 enzymes, reducing the production of prostaglandins that contribute to the peripheral and central sensitization of inflammatory pain (10). COX-1 is physiologically active throughout the body, while COX-2, in addition to some physiological functions, mediates the elevation of prostaglandins, associated with inflammation, pain, and fever (11).

Among the drugs used preemptively to control postoperative pain in third molar surgery are diclofenac (12), ketoprofen (13), lornoxicam (8), and ibuprofen (14,15). Ibuprofen is a non-selective inhibitor of COX-1 and COX-2, which inhibits the formation of inflammatory mediators via the arachidonic acid cascade (16).

The preemptive use of intravenous (IV) ibuprofen to reduce postoperative pain in third molar surgery has been the subject of recent studies (17,18). However, there is no consensus regarding its use in clinical practice. Thus, the present study aimed to evaluate the effect of preemptive IV ibuprofen on postoperative pain in lower third molar extractions.

## METHODS

### Protocol and registration

The present systematic review was registered in the PROSPERO database (http://www.crd.york.ac.uk/PROSPERO, PROTOCOL: CRD42020210477) and performed according to the Preferred Reporting Items for Systematic Review and Meta-Analysis (PRISMA) guidelines (http://www.prisma-statment.org) (19) and the Joanna Briggs Institute (JBI) guidelines (20).

### Study design and eligibility criteria

This systematic review was based on the research question based on the PICO acronym, described as follows: “Is the use of preemptive IV ibuprofen (Intervention) more effective in reducing pain (Outcome) when compared to placebo (Comparison) in patients undergoing lower third molar surgery (Population)?”

The inclusion criteria consisted of randomized controlled trials that compared the preemptive use of IV ibuprofen in patients undergoing extraction of the lower third molars. There were no year- or language-related restrictions. Regarding the degree of difficulty of extraction, there was no restriction in the Pell and Gregory classification, in which the teeth are classified for the ascending mandibular ramus and depth of inclusion in the mandible (21); no relation to Winter classification, which assesses the positioning of the third molar relative to the long axis of the lower second molar (22); or even the type of inclusion (bone, sub-mucous, and semi-included).

The exclusion criteria were as follows: 1) studies with patients undergoing extraction of primary teeth; 2) studies without a control group; 3) letters to the editor or editorials; 4) meeting abstracts; and 5) personal opinions, books, and/or book chapters.

### Sources of information, search, and selection of studies

The search was conducted in May 2020 and updated in March 2021. Seven databases (MEDLINE [*via* PubMed], Embase, Web of Science, Latin American and Caribbean Literature in Health Sciences, Cochrane, Scielo, and LIVIVO), and two citation databases (Scopus and Web of Science) were used as primary search sources. The Open Gray and Open Thesis databases were used to partially capture the “grey literature.” A manual search was also performed through a systematic analysis of the references of the eligible articles. These steps were performed to minimize selection and publication bias.

Medical Subject Headings, Health Sciences Descriptors, and Emtree (Embase Subject Headings) resources were used to select search descriptors. The Boolean operators “AND” and “OR” were used to improve the search strategy through various combinations. The search strategies are presented in Table 1.

The studies were selected in four stages. In the first stage, studies were identified after bibliographic research in the databases. The results obtained were exported to EndNote Web™ software (Thomson Reuters, Toronto, Canada), in which duplicates were removed. The remaining results were exported to Microsoft Word™ 2019 (Microsoft ™ Ltd, Washington, USA), and duplicate articles were manually removed.

Before the second stage, a calibration exercise was performed before the selection of the studies, in which the reviewers discussed the eligibility criteria. In the second stage, a thorough analysis of the study titles was performed. The abstracts were read for the initial application of the eligibility criteria (third stage). In the fourth stage, the preliminary eligible studies had their full texts obtained and evaluated to verify whether they met the eligibility criteria.

### Data collection

A calibration exercise was performed with the two reviewers to ensure consistency between them, in which the information was extracted from an eligible study. After the selection, the studies were analyzed by two reviewers, who extracted the following information from the articles: study identification (author, year, country, and place of research); ethical parameters and use of the consent form for research participants; use of the CONSORT checklist; registration in clinical trials; sample characteristics (study groups, sex, time of preoperative medication administration, postoperative medication schedule, Winter classification, Pell & Gregory, type of inclusion [bone, sub-mucous, and semi-included], tool used for pain assessment); and results (patients excluded, complications, patients who did not return, lost to follow-up, an anesthetic solution used, total loss, and average pain assessment).

### Risk of individual study bias

The JBI Critical Assessment Tool for use in JBI Systematic Reviews for randomized controlled trials (23) was used to assess the risk of bias in the studies selected. Two authors independently assessed each domain relative to the potential risk of bias, as recommended by the PRISMA statement (19).

Each study was categorized according to the percentage of positive responses to the questions corresponding to the assessment instrument. The risk of bias was considered high when the study obtained 49% of the answers “yes,” moderate when the study obtained 50-69% of the answers “yes,” and low when the study reached more than 70% of the answer “yes.”

### Summary measures and summary of results

The performance of the studies was measured by evaluating the treatment protocol according to preoperative and postoperative medications, time to assess pain levels, and the use of analgesics during the postoperative period. Pain levels were assessed using a visual analog scale (VAS). For continuous results, descriptive statistics such as mean differences and standard deviations were used to summarize the data from the included studies.

The primary outcome of this study was the assessment of pain levels when using ibuprofen intravenously. The final absolute sample size of the patients who underwent extraction of the third molars was established. A meta-analysis was not justified owing to a large amount of clinical, statistical, and methodological heterogeneity.

## RESULTS

### Study selection

**Figure 1:**
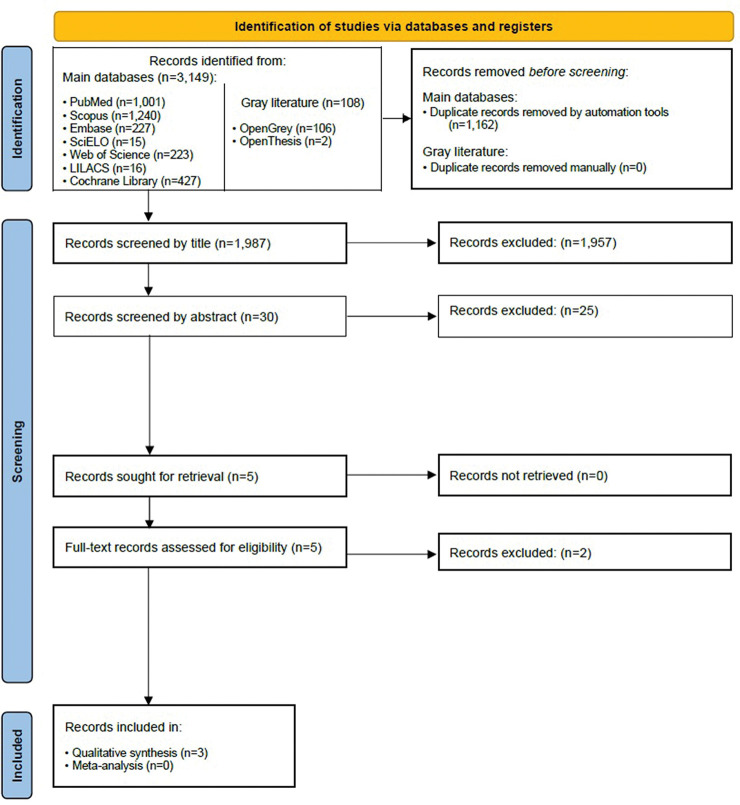
Preferred Reporting Items for Systematic Review and Meta-Analysis (PRISMA) 2020 flow diagram.

During the first phase of the study selection, 3,257 results were found distributed in nine electronic databases, including the “grey literature.” After removing duplicates, 1,987 articles were retained for the analysis of titles and abstracts. After reading the titles and abstracts, five studies evaluated the full texts. After a full reading, two studies were eliminated for the following reasons: one study proposed to assess the presence of trismus and edema without pain assessment using the VAS (18); however, the study by Viswanath et al. (17) did not have a placebo group. The references of the three potentially eligible studies were carefully evaluated, adding up to a total of 79 articles. No additional articles were selected, resulting in three studies for the qualitative analysis. Figure 1 illustrates the search, identification, inclusion, and exclusion process of the articles.

### Characteristics of eligible studies

The studies were published in 2019 and conducted in Turkey (7,24,25). All studies (7,24,25) respected the ethical criteria for the development of the recommended research in the country of origin, applying a consent term for all volunteers participating in the study. Only two studies (7,24) used CONSORT as a methodological guideline, and two studies (24,25) provided the clinical trial registration number.

A total of 150 treated patients were included, with 75 treated using IV ibuprofen and 75 in the control group. The age of the patients in each study ranged from 18 to 35 years. All studies (7,24,25) used the VAS to assess postoperative pain.

Degirmenci and Yalcin (24) referred to the classification of Pell and Gregory (21) as II-B. Demirbas et al. (7) referred to surgical extraction of the lower third molar with bone impaction, following the Winter classification (horizontal or mesioangular position) (22). Küpeli and Gülnahar (25) referred to third-angled or horizontal molars. Three studies (7,24,25) were performed on third molars with a similar challenge. The three studies cited the anesthetic solutions used to induce local anesthesia. Two studies used articaine as an anesthetic salt (7,24) differing in the concentrations of vasoconstrictors of 1:100,000 and 1:200,000 epinephrine. Küpeli and Gülnahar (25) used lidocaine associated with 1:100,000 epinephrine.

Regarding postoperative medications, in the study by Degirmenci and Yalcin (24), all participants received antibiotic therapy (amoxicillin-clavulanate 875/125 or 300 mg of clindamycin)+paracetamol 500 mg. In the study by Demirbas et al. (7), participants received acetaminophen 500 mg (paracetamol) as a rescue medication. The study by Küpeli and Gülnahar (25) used an infusion of dexketoprofen+methylprednisolone+sultamicillin tosylate. Only two studies mentioned the use of analgesics in the postoperative period (7,24). Only one study recorded mean arterial pressure and heart rate in the preoperative period (25).

More details on the characteristics of the studies are shown in Table 2.

### Risk of bias

Two eligible studies had a “low” risk of bias (24,25), while one study had a “moderate risk” (7). Table 3 shows detailed information on the risk of individual bias in the included studies (23). Item 1 was marked as “unclear” in a study as the randomization method was not described (25). Item 2 was marked as “unclear” in another study as it was not clear whether the allocation of treatment groups was hidden (7). Item 5 was marked as “unclear” in a study as it was not clear whether the person administering the treatment was blind to the treatment assignment (7). Item 6 was marked as “unclear” in two studies as the randomization blinding method was not explicit (7,24). Item 9 was marked as “not applicable” in one study, as there were no losses, and the entire sample was analyzed (24). Item 9, in the study by Küpeli and Gülnahar (25), was marked as “unclear.” Item 13 was marked as “unclear” in two studies, which showed dispersion and loss between the groups; however, the study design did not mention this fact (7,25).

### Individual study results

Degirmenci and Yalcin (24) assessed postoperative pain at 1h/1h in the first 12h and followed up until the sixth postoperative day. Demirbas et al. (7) assessed postoperative pain in the first 24h after surgery. Küpeli and Gulnahar (25) recorded pain in the first 25 min, at the end of the surgery, and in the first 48h after surgery.

All studies used pain analysis methods using VAS. To facilitate pain assessment over time, it was systematically summarized in three periods: day 0 (up to 24h immediately after surgery), day 1 (24-48h after surgery), and day 2 (48-72h after surgery). Degirmenci and Yalcin (24) assessed the average postoperative pain for the first 72h after the procedure. However, Demirbas et al. (7) assessed only the values of the first 24h, and Küpeli and Gülnahar (25) did not express the numerical averages in the text.

In the study by Degirmenci and Yalcin (24), the authors found no difference between the variables of pain, analgesic consumption, and the first rescue of the analgesic between IV ibuprofen and IV placebo after 72h. In the study by Demirbas et al. (7), the pain record was statistically higher in the IV placebo group than in the preoperative IV ibuprofen group, and analgesic consumption was higher in the placebo group in the first 24h. In the study by Küpeli and Gülnahar (25), pain scores were significantly higher in the placebo group than in the preemptive IV ibuprofen group 1-4h postoperatively; however, without statistical difference after 48h. Küpeli and Gülnahar (25) did not record analgesic consumption.

Other results common to two or more studies are shown in Table 4.

## DISCUSSION

Several medications have been used and tested as preemptive medications, such as steroidal anti-inflammatory drugs (26,27), NSAIDs (3), and opioids (28). The use of NSAIDs in preemptive analgesia aims to prevent the release of neurotransmitters and inflammatory mediators (29). Oral ibuprofen is among the most commonly used NSAIDs for preemptive analgesia in the third molars (14). The IV form of ibuprofen was approved for the management of mild and moderate to severe pain in adults as an adjunct to opioid analgesics (30). Studies with IV ibuprofen support its efficiency in the treatment of various types of pain of surgical and non-surgical origin (31) and the reduction of postoperative pain in the third molars, as shown in the eligible studies in the present review (7,24,25).

In the extraction of third molars, the location of the tooth, depth of impaction, angulation, and root morphology are associated with the degree of surgical difficulty (32). The occurrence of pain, trismus, and edema in the postoperative period of third molar surgery is associated with age, sex, body mass, time of surgery, and tissue trauma, such as odontosection and osteotomy (33). Additionally, the soft tissue plays an important role in postoperative pain. A study by Patel et al. (34) on different types of flaps in the postoperative pain of third molar surgery concluded that pain was not generated due to the incision itself; however, due to the release of mediators, such as bradykinin, serotonin, and prostaglandins. In the eligible studies (7,24), patients whose surgery duration exceeded 30 and 40 min were excluded from the study. Küpeli and Gülnahar (25) did not refer to the surgical time. The absence of surgical time is a selection bias that must be considered.

The extraction of the lower third molar is considered a good clinical model for assessing preemptive analgesia (3,35). After the extraction of the third molar, inflammatory mediators, such as prostaglandins, bradykinin, histamine, and leukotriene are released (36,37). Ibuprofen inhibits COX-1 and COX-2, leading to inhibition of prostaglandin synthesis by decreasing the conversion of arachidonic acid (30). A clinical study conducted on gingival tissue during the extraction of lower third molars using a preemptive dose of 400 mg ibuprofen, observed the expression of COX-1 and COX-2 genes in the placebo group, and there was a reduction of COX-2 in the ibuprofen group (38). IV ibuprofen is a non-selective inhibitor of COX-1 and COX-2 (39).

The level of postoperative pain was assessed in previous studies (7,24,25) using the VAS. This scale is a simple method used to assess the variations in pain intensity (40). Studies show that the VAS is a highly reliable instrument for measuring acute pain (41). Pain is subjective and may vary according to sex, age, and previous painful experiences (42).

In the study by Degirmenci and Yalcin (24), the authors found no difference in pain scores assessed by the VAS between IV ibuprofen and IV placebo. Demirbas et al. (7) reported higher pain in the IV placebo group than that in the preoperative IV ibuprofen group. This result agrees with the study by Küpeli and Gülnahar (25), who concluded that preemptive IV ibuprofen proved to be efficient even alone. The plasma concentration of IV ibuprofen is twice as high as oral ibuprofen, reaching a maximum concentration in 0.11h, while oral ibuprofen reached a maximum in 1.5h (43). Bergese et al. (31) evaluated pain by different etiologies, n which patients received 800 mg of IV ibuprofen every six hours, and a decrease in VAS scores was observed in the six hours following the medication. The pharmacokinetic profile of IV ibuprofen 800 mg (infusion for 30 min), 400 mg (infusion for 5 to 10 min), and ibuprofen 400 mg (oral) showed that the oral dose could not reach the maximum plasma concentration level of any IV dose (44). Oral administration shows bioavailability, which is usually reduced by the effect of the first hepatic passage.

One of the parameters for assessing the preemptive effects of medications is the consumption of supplementary postoperative analgesia and the time interval for the first analgesic (45). Degirmenci and Yalcin (24) showed a difference in analgesic consumption and the time of the first analgesic rescue between IV ibuprofen and IV placebo. Demirbas et al. (7) showed that the IV ibuprofen group, in the first hour after surgery, did not use postoperative analgesia and consumed a lower average dose in the first 24h. Küpeli and Gülnahar (25) did not mention the rescue of postoperative medications. In the present review, the preemptive effect of IV ibuprofen appeared to generally reduce acute pain and the number of postoperative episodes. Such findings were corroborated by Viswanath et al. (17), who reported that patients receiving preemptive 800 mg of IV ibuprofen in the extraction of third molars showed lower pain scores and opioid consumption than that with 1000 mg of IV acetaminophen. IV ibuprofen may have multimodal analgesic effects when combined with a central and peripheral nerve block (46,47).

In the eligible studies (7,24,25), lidocaine and articaine associated with a vasoconstrictor were used as local anesthetics. According to Liporaci (48), local anesthesia is preemptive analgesia, which inhibits the sensation of pain during the first few hours after surgery. The local anesthetic used in preemptive analgesia studies can be considered a confounding factor in the results (48). In the eligible studies, the action of anesthetic salts had an intermediate duration and reduced action on analgesia.

Among the limitations of this systematic review is the small number of eligible studies on the preemptive effect of IV ibuprofen on the extraction of third molars. The main reason for this is that the protocol is new. Additionally, it is an unusual route of administration on an outpatient basis. The oral route is more accepted by patients and does not require professional training. The existence of different types of preemptive protocols, heterogeneity of groups, and representation of the data did not allow a statistical analysis of the results, making it impossible to perform the meta-analysis. However, the present study is original and has the important positive point of a low risk of bias of the selected articles, which provides safer and more reliable results. The preemptive IV ibuprofen proved to be efficient in reducing pain and postoperative analgesic consumption, representing a good alternative in preemptive analgesia. Further randomized clinical studies should be conducted to observe the methodological quality to establish itself as a clinical protocol.

## CONCLUSION

Based on the results of the eligible studies, it is possible to suggest that the use of preemptive IV ibuprofen in the extraction of third molars reduces pain and analgesic consumption after the surgical procedure. However, clinical studies should be encouraged to provide more scientific evidence on this subject.

## AUTHOR CONTRIBUTIONS

Silva PUJ, Meneses-Santos D and Silva RP had complete roles in the identification, manuscript review, data extraction, quality assessment, analysis, manuscript drafting and revision. Silva RP conceived the idea. Vieira WA, Ramacciato JC, Silva MCP, Rode SM and Paranhos LR played major roles in the analysis, manuscript drafting and revision. All authors have read and approved the final version of the manuscript for publication. All authors agreed to be equally accountable for all aspects of this study.

## Figures and Tables

**Table 1:** Strategies for database search.

Database	Search strategy
Medline (via PubMed) http://www.ncbi.nlm.nih.gov/pubmed	**#1** ("Tooth Extraction*"[MeSH Terms] OR "Tooth Extraction*"[Title/Abstract] OR "Extraction, Tooth"[Title/Abstract] OR "Extractions, Tooth"[Title/Abstract] OR "Oral Surgical Procedures"[MeSH Terms] OR "Oral Surgical Procedures"[Title/Abstract] OR "Surgical Procedures, Oral"[Title/Abstract] OR "Maxillofacial Procedure*"[Title/Abstract] OR “Third molar”[Title/Abstract] OR “Molar, Third”[MeSH Terms] OR “Molar, Third”[Title/Abstract])
	**#2** ("Ibuprofen"[MeSH Terms] OR "Ibuprofen"[Title/Abstract] OR "Anti-Inflammatory Agent*"[MeSH Terms] OR "Anti-Inflammatory Agent*"[Title/Abstract] OR "Antiinflammatories"[Title/Abstract] OR "Anti-Inflammatories"[Title/Abstract] OR "Anti-Inflammatory Agents, Non-Steroidal"[MeSH Terms] OR "Anti-Inflammatory Agents, Non-Steroidal"[Title/Abstract] OR "NSAIDs"[Title/Abstract] OR "Intravenous Ibuprofen"[Title/Abstract] OR "Cyclooxygenase Inhibitors"[Title/Abstract] OR "Cyclooxygenase Inhibitors"[MeSH Terms])
	**#1 AND #2**
Scopus http://www.scopus.com/	**#1** (TITLE-ABS-KEY ("Tooth Extraction*")) OR (TITLE-ABS-KEY ("Extraction, Tooth")) OR (TITLE-ABS-KEY ("Extractions, Tooth")) OR (TITLE-ABS-KEY ("Oral Surgical Procedures")) OR (TITLE-ABS-KEY ("Surgical Procedures, Oral")) OR (TITLE-ABS-KEY ("Maxillofacial Procedure*")) OR (TITLE-ABS-KEY ("Third molar")) OR (TITLE-ABS-KEY ("Molar, Third"))
	**#2** (TITLE-ABS-KEY ("Ibuprofen")) OR (TITLE-ABS-KEY ("Anti-Inflammatory Agent*")) OR (TITLE-ABS-KEY ("Antiinflammatories")) OR (TITLE-ABS-KEY ("Anti-Inflammatory Agents, Non-Steroidal")) OR (TITLE-ABS-KEY ("Anti-Inflammatories")) OR (TITLE-ABS-KEY ("NSAIDs")) OR (TITLE-ABS-KEY ("Intravenous Ibuprofen")) OR (TITLE-ABS-KEY ("Cyclooxygenase Inhibitors"))
	**#1 AND #2**
Web of Science http://apps.webofknowledge.com/	**#1** TS=("Tooth Extraction*") OR TS=("Extraction, Tooth") OR TS=("Extractions, Tooth") OR TS=("Oral Surgical Procedures") OR TS=("Surgical Procedures, Oral") OR TS=("Maxillofacial Procedure*") OR TS=("Third molar") OR TS=("Molar, Third")
	**#2** TS=("Ibuprofen") OR TS=("Anti-Inflammatory Agent*") OR TS=("Antiinflammatories") OR TS=("Anti-Inflammatory Agents, Non-Steroidal") OR TS=("Anti-Inflammatories") OR TS=("NSAIDs") OR TS=("Intravenous Ibuprofen") OR TS=("Cyclooxygenase Inhibitors")
	**#1 AND #2**
Embase https://www.embase.com	**#1** 'tooth extraction':ab,ti AND [embase]/lim OR 'oral surgery':ab,ti AND [embase]/lim OR 'maxillofacial procedure':ab,ti AND [embase]/lim OR 'third molar':ab,ti AND [embase]/lim
	**#2** 'ibuprofen':ab,ti AND [embase]/lim OR 'antiinflammatory agent':ab,ti AND [embase]/lim OR ‘antiinflammatories’:ab,ti AND [embase]/lim OR 'nonsteroid antiinflammatory agent':ab,ti AND [embase]/lim OR 'anti inflammatories':ab,ti AND [embase]/lim OR 'nsaids':ab,ti AND [embase]/lim OR ‘intravenous ibuprofen’:ab,ti AND [embase]/lim OR 'prostaglandin synthase inhibitor':ab,ti AND [embase]/lim
	**#1 AND #2**
LILACS http://lilacs.bvsalud.org/	**#1** ("Tooth Extraction" OR "Extraction, Tooth" OR "Extractions, Tooth" OR "Tooth Extractions" OR "Oral Surgical Procedures" OR "Surgical Procedures, Oral" OR "Maxillofacial Procedure" OR "Maxillofacial Procedures" OR “Third molar” OR “Extração de terceiro molar” OR “Terceiro molar” OR “Extração Dentária” OR “Extracción Dental” OR “Dente Serotino” OR “Tercer Molar”)
	**#2** ("Ibuprofen" OR "Anti-Inflammatory Agents" OR "Anti Inflammatory Agents" OR "Antiinflammatories" OR "Anti-Inflammatories" OR "Non-Steroidal Anti-Inflammatory Agents" OR "NSAIDs" OR "Intravenous Ibuprofen" OR "Cyclooxygenase Inhibitors" OR “Ibuprofeno” OR “Anti-Inflamatórios” OR “Antiinflamatorios” OR “Anti-Inflamatórios não Esteroides” OR “Antiinflamatorios no Esteroideos”)
	#3 (db:("BBO" OR "LILACS"))
	**#1 AND #2 AND #3**
Scielo http://www.scielo.org/	**#1** (Tooth Extraction) OR (Extraction, Tooth) OR (Oral Surgical Procedures) OR (Maxillofacial Procedure) OR (Third molar) OR (Extração de terceiro molar) OR (Terceiro molar) OR (Extração Dentária) OR (Extracción Dental) OR (Dente Serotino) OR (Tercer Molar)
	**#2** (Ibuprofen) OR (Anti-Inflammatory Agents) OR (Anti Inflammatory Agents) OR (Antiinflammatories) OR (Anti-Inflammatories) OR (Non-Steroidal Anti-Inflammatory Agents) OR (NSAIDs) OR (Intravenous Ibuprofen) OR (Cyclooxygenase Inhibitors) OR (Ibuprofeno) OR (Anti-Inflamatórios) OR (Antiinflamatorios) OR (Anti-Inflamatórios não Esteroides) OR (Antiinflamatorios no Esteroideos)
	#1 AND #2
Cochrane Library http://www.cochranelibrary.com/	**#1** MeSH descriptor: [Tooth Extraction] this term only **#2** MeSH descriptor: [Oral Surgical Procedures] this term only **#3** MeSH descriptor: [Molar, Third] explode all trees **#4** ("third molar"):ti,ab,kw **#5** (Maxillofacial Procedure):ti,ab,kw **#6** #1 OR #2 OR #3 OR #4 OR #5
	**#7** MeSH descriptor: [Ibuprofen] explode all trees **#8** MeSH descriptor: [Anti-Inflammatory Agents] explode all trees **#9** MeSH descriptor: [Anti-Inflammatory Agents, Non-Steroidal] this term only **#10** MeSH descriptor: [Cyclooxygenase Inhibitors] this term only **#11** ("Intravenous Ibuprofen"):ti,ab,kw **#12** #7 OR #8 OR #9 OR #10 OR #11
	**#6 AND #12**
OpenGrey http://www.opengrey.eu/	(“Tooth Extraction” OR “Oral Surgical Procedures” OR “Maxillofacial Procedure” OR “Ibuprofen”)
OpenThesis http://www.openthesis.org/	("Tooth Extraction" OR "Oral Surgical Procedures" OR "Maxillofacial Procedure") AND ("Ibuprofen" OR "Anti Inflammatory Agents")

MeSH, Medical Subject Headings.

**Table 2:** Summary of the main characteristics of the eligible studies.

Author, year	Age (years)	Total number of patients	Sex	Groups	Time of medication administration	Postoperative medication	Excluded patients	Complications	Patients who did not return
Degirmenci, and Yalcin (24)	18-24	40	Group 1 M: 6 F: 14 Group 2 M: 4 F:16	Group 1: oral placebo+intravenous placebo Group 2: oral placebo+intravenous ibuprofen 400 mg	60 min before and infusion 30 min before	Group 1: paracetamol 500 mg every 8h for 6 days Group 2: paracetamol 500 mg every 8h for 6 days	G 1: 2 G 2: 2	G 1: 2 G 2: 1	G 1: -- G 2: --
Demirbas et al. (7)	Mean: 25.1	50	--	Group 1: 800 mg of intravenous ibuprofen 60 min before surgery and intravenous placebo (100 mL of saline) 60 min after surgery Group 3: intravenous placebo (100 mL of saline) 60 min before and after surgery	60 min before surgery	Acetaminophen 500 mg	G 1: 0 G 3: 0	G 1: -- G 3: --	G 1: 0 G 3: 0
Küpeli and Gülnahar (25)	20-35	40	Group 2 M: 8 F: 12 Group 3 M:8 F:12	Group 2: preoperative intravenous infusion including Ibuprofen 800 mg for 30 min Group 3: preoperative intravenous infusion of placebo (normal saline) for 30 min	15 min before surgery	Infusion of dexketoprofen+methylprednisolone (Prednol-L; Mustafa Nevzat, Istanbul, Turkey) 40 mg+sultamicillin sulfate (Ampisid; Mustafa Nevzat) in 150 mL of normal saline	G 2: -- G 3: --	G 2: -- G 3: --	G 2: -- G 3: --

M, male; F, female; G 1, group 1; G 2, group 2; G 3, group 3; --, not mentioned in the manuscript; Paracetamol = Acetaminophen.

**Table 3:** Risk of bias assessed by the Joanna Briggs Institute (JBI) Critical Appraisal Tools for use in JBI Systematic Reviews for randomized controlled trial studies (23).

Eligible articles	Q.1	Q.2	Q.3	Q.4	Q.5	Q.6	Q.7	Q.8	Q.9	Q.10	Q.11	Q.12	Q.13	% yes/risk
Degirmenci and Yalcin (24)	√	√	√	√	√	U	√	√	--	√	√	√	√	84.6%/ low risk
Demirbas et al. (7)	√	U	√	√	U	U	√	√	√	√	√	√	U	69.2%/ moderate risk
Küpeli and Gülnahar (25)	U	√	√	√	√	√	√	√	U	√	√	√	U	76.9%/ low risk

Q.1: Was true randomization used to assign participants to treatment groups? Q.2: Was the allocation to treatment groups concealed? Q.3: Were treatment groups similar at baseline? Q.4: Were the participants blind to the treatment assignment? Q.5: Were those who deliver treatment blind to treatment assignment? Q.6: Were outcome assessors blinded to treatment assignment? Q.7: Were treatment groups treated identically other than the intervention of interest? Q.8: Was follow-up complete, and if not, were differences between groups in terms of their follow-up adequately described and analyzed? Q.9: Were the participants analyzed in the groups to which they were randomized? Q.10: Were the outcomes measured equally for the treatment groups? Q.11: Were the outcomes measured reliably? Q.12:Was appropriate statistical analysis used? Q.13: Was the trial design appropriate and any deviations from the standard randomized controlled trials design (individual randomization, parallel groups) accounted for in the performance and analysis of the trial? / √, Yes; --, No; U, Uncertain; N/A, Not applicable.

**Table 4:** Summary of the main qualitative/quantitative characteristics of the eligible studies.

Author, year	Lost to follow-up	Total number of patients	Pain assessment: mean			Postoperative analgesic consumption (time to the first rescue)	Conclusion
			Day (24h)	Day (48h)	Day (72h)		
Degirmenci and Yalcin (24)	G 1: 2 G 2: 0	G 1: 20 G 2: 20	G1:36.75* G2:35.37*	G1:37.5 G2: 30	G1:31.5 G2: 10	G1: Seven analgesics (240 min) G2: Eight analgesics (240 min)	The study found no difference in the classification of pain and time to first analgesic rescue among the groups evaluated.
Demirbas et al. (7)	G 1: 0 G 3: 0	G 1: 25 G 3: 25	G1:14.5% G3:42.6%	-- --	-- --	G1: 640 mg (in 24h) G3: 1840 mg (in 24h)	Preemptive use of IV Ibuprofen decreases the experience of pain and requirement for rescue analgesia within the first 24 h after surgery.
Küpeli and Gülnahar (25)	G 2: 0 G 3: 0	G 2: -- G 3: --	G2: -- G3: --	-- --	-- --	G 2: -- G 3: --	Pain scores were higher in the placebo group than in the Ibuprofen group alone.

G 1: group 1; G 2: group 2; G 3: group 3; IV: intravenous; * arithmetic average; --: the absolute value or average was not expressed in the manuscript.
